# Cardiovascular system in larval zebrafish responds to developmental hypoxia in a family specific manner

**DOI:** 10.1186/1742-9994-3-4

**Published:** 2006-03-15

**Authors:** Francisco B-G Moore, Michelle Hosey, Brian Bagatto

**Affiliations:** 1 Department of Biology, University of Akron, Akron, Ohio, USA

## Abstract

**Background:**

Genetic and environmental variation are both known to influence development. Evolution of a developmental response that is optimized to the environment (adaptive plasticity) requires the existence of genetic variation for that developmental response. In complex traits composed of integrated sets of subsidiary traits, the adaptive process may be slowed by the existence of multiple possible integrated responses. This study tests for family (sibship) specific differences in plastic response to hypoxia in an integrated set of cardiovascular traits in zebrafish.

**Results:**

Cardiac output, which is the integrated product of several subsidiary traits, varied highly significantly between families, and families differed significantly in the degree and direction of response to developmental oxygen level. The cardiac output response to oxygen environment was entirely family specific with no significant overall trend due to oxygen level. Constituent physiological variables that contribute to cardiac output all showed significant family specific response to hypoxia. Traits that were not directly related to cardiac output, such as arterial and venous diameter, and red blood cell velocities did not respond to hypoxia in a family specific manner.

**Conclusion:**

Zebrafish families vary in their plastic response to hypoxia. Genetic variation in plastic response to hypoxia may therefore provide the basic ingredient for adaptation to a variable environment. Considerable variation in the degree of familial response to hypoxia exists between different cardiovascular traits that may contribute to cardiac output. It is possible that the integration of several subsidiary traits into cardiac output allows the maintenance of genetic variance in cardiac response.

## Background

What processes constrain and redirect the outcome of the adult phenotype? Many developmental studies at all biological levels attempt to answer this question. While genetic instructions provide the basic template for development, the ultimate phenotype also depends on environmental influences [1-3]. Recent explorations of developmental physiology in fish [4], birds [5], amphibians [6], reptiles [7,8] and mammals [9] have begun to separate the influences of genetics and environment on physiology. Such studies suggest that within a given environment, developmental templates for a given family (i.e. sibling group) include unique instructions that direct physiological change throughout development. Both environment and genetics appear to influence physiological variation, but genetics and environment can interact such that different genotypes will react differently to the same environmental change. This study tests for this type of family specific difference in the manner in which embryos respond to environmental stressors. If such family specific differences exist, they may provide the raw material for adaptation of developmental response.

Developmental plasticity, in which juvenile environment shapes adult phenotype, is common [3] and may increase fitness in some cases. If a single genotype can develop into different specialist phenotypes for each of a number of different environments, then it may out-compete generalist phenotypes in each environment. However, the evolution of such adaptively plastic phenotypes requires genetic variation for plastic response. Plasticity and the genetic basis of plasticity are important topics in evolutionary biology because they may help explain adaptive constraints and tradeoffs [3,10-12] as well as mechanisms of phenotypic integration [13]. Despite a great deal of progress in understanding plasticity, the degree to which plastic responses are adaptive, and the constraints on the evolution of plastic development remain critical areas of research [14]. Of special interest is the role of plasticity in the integration of phenotype which has rarely been studied [13]. If the phenotypic response to a variable environment involves complex interactions, as is expected in many physiological traits, then adaptive plasticity may be highly constrained because a balance of different plastic responses in several traits is required. This is because, although selection may increase the frequency of the most fit phenotype in a given generation, a number of different genotypes can all produce highly fit phenotypes. Recombination between these high fitness phenotypes will result in the production of offspring of unpredictable phenotype in the next generation.

Detection of developmental plasticity is evolutionarily important when the environmental parameters are within the range of normal environmental variation and of a magnitude that they provide significant stress to the organism. In these cases, a developmental response may have evolved that buffers the effects of environmental variation. For example, many species of fish routinely experience mild ([O_2_] < 5 mg l^-1^) to severe ([O_2_] < 1 mg l^-1^) hypoxia that triggers a suite of organismal, cellular, and genetic responses. In many cases, these responses are attenuated as the hypoxic exposure becomes chronic. Chronic hypoxia has a profound effect on fish behaviour [15], many aspects of cardiovascular physiology [16], and even general morphology [17]. Larval fish are often incapable of avoiding hypoxic zones and hence may experience severely altered or even arrested development [18]. At this time, there are very few studies that test the prolonged influence of developmental hypoxia in fish, and there are no studies to our knowledge that test family specific cardiovascular responses to developmental hypoxia. For all of these reasons, hypoxia during development in fish is an ideal test for family specific developmental plasticity.

We chose to use a simple quantitative genetic design to investigate family specific response to developmental stress in zebrafish (*Danio rerio*). This species can commonly be found in fast moving river tributaries, slow moving and stagnant bodies of water [19] so that larvae are likely to see considerable variation in oxygen environment. While even severe hypoxia may not in many cases increase larval mortality directly (unpublished data), hypoxia has been shown to cause substantial changes in development rate [20] and other fitness related traits [15].

We bred females and males from a common environment to produce sets of full sibling eggs to test for variation between families in developmental physiology. Each family was evenly split into two groups. One group was reared in normoxia and the other in chronic hypoxia, allowing us to test for influence of developmental environment on integrated cardiovascular development. We compared cardiovascular anatomy and physiology of developing zebrafish in hypoxic and normoxic water by family. We hypothesized that developmental response to the oxygen environment would be family specific. We tested for family specific developmental response across 12 different physiological and morphological traits related to cardiovascular function.

## Results

### Vessel anatomy

**Figure 1:**
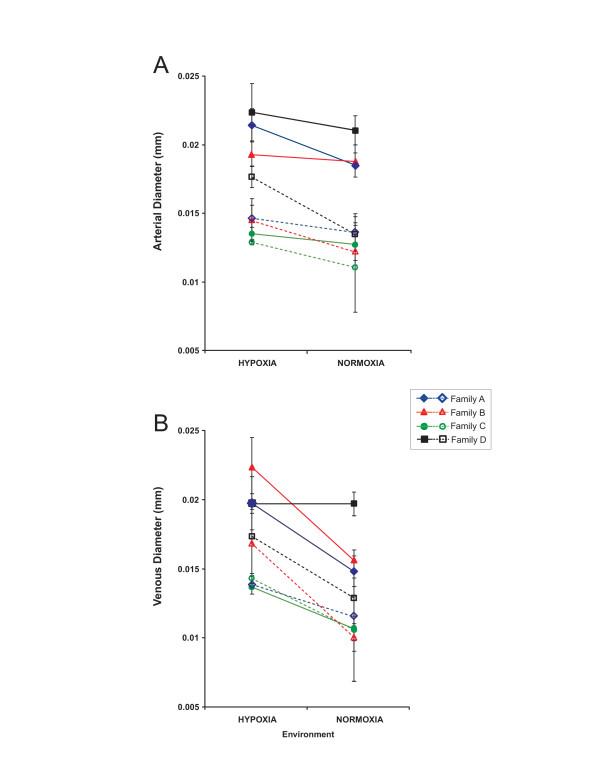
**Mean vessel diameter by family and environment**. A) Family mean arterial diameter in mm for larvae reared in normoxic and hypoxic environments. Solid lines and symbols represent means for dorsal aorta. Intermittent lines and open symbols represent means for caudal arteries. B) Venous diameter in mm for larvae reared in each environment. Solid line and symbols represent means for dorsal vein. Intermittent lines and open symbols represent veins. Error bars represent one standard error. Significant variation exists between families for venous diameter, while significant variation between environments in arterial diameter.

The developmental oxygen environment was a significant source of variation in two arterial diameters but not in either of the two venous diameters (Table 1). In both arteries measured, hypoxia-reared individuals from a given family had larger vessel diameters than their normoxia-reared siblings (Figure 1). In both the dorsal artery and dorsal vein, family was a significant source of variation (Table 1) largely due to the low vessel diameter in family C (Figure 1A). There was no interaction between developmental environment and family for any measured vessel diameter indicating that no family specific influence of environment exists on vessel diameter.

**Table 1:** Sources of variation in blood vessel diameter for four major vessels. ANOVA models were run using Family, Environment and Family by Environment interaction as the modelled sources of variation. Critical F values for the given degrees of freedom (DF) and the calculated F values are given for diameters of the Dorsal and Caudal veins and the dorsal and Caudal arteries. Significant F values (p < 0.05) are in bold face and asterisks represent highly significant (p < 0.005) p values.

Source	DF	F value				
		
		Critical	Dorsal vein	Caudal vein	Dorsal artery	Caudal artery
Family	3	2.91	11.35*	1.35	8.74*	0.58
Environment	1	4.16	1.87	3.70	12.10*	7.71*
Family × Environment	3	2.91	0.30	0.30	2.11	0.46
Error	38					

### Red blood cell velocity

**Figure 2:**
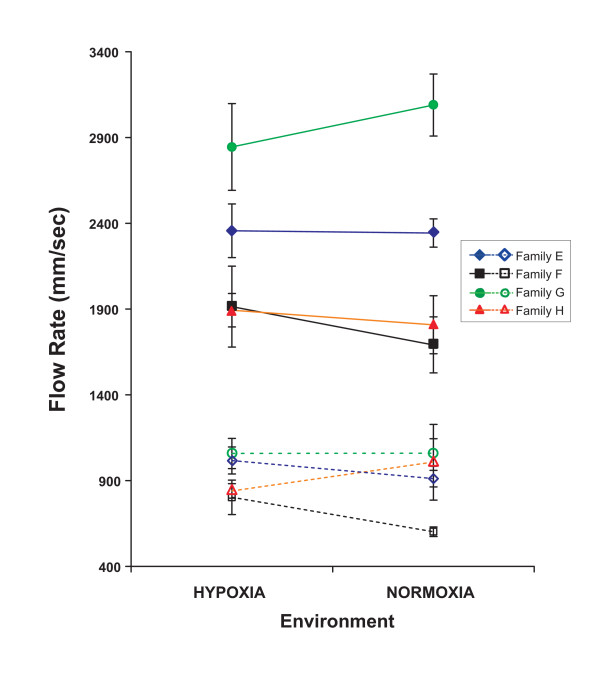
**Mean red blood cell velocity by family and environment**. A) Family mean arterial red blood cell flow rate is presented for larvae reared in normoxic and hypoxic environments. B) Family mean venous (open symbols) red blood cell flow rate is presented for larvae reared in normoxic and hypoxic environments. Error bars represent one standard error. Environment was not a significant source of variation in flow rate, while family was a significant source of variation in both arterial and venous flow rates.

Zebrafish family was a significant source of variation in both dorsal artery and dorsal vein red blood cell velocity. Developmental oxygen level was not a significant source of variation in red blood cell velocities either directly or through an interaction with family (Table 2). Red blood cell velocity in the dorsal artery tended to mirror red blood cell velocity in the corresponding vein (Figure 2) in that no families showed a significant difference across treatments (for all T-tests, p was less than 0.05).

**Table 2:** Sources of variation in red blood cell velocity. ANOVA models were run using Family, Environment and Family by Environment interaction as the modelled sources of variation. Critical F values for the given degrees of freedom (DF) and the calculated F values are given for Red Blood Cell Velocity in the dorsal vein and artery. Significant F values (p < 0.05) are in bold face and asterisks represent highly significant (p < 0.005) p values.

Source	DF	F value		
		
		Critical	Venous velocity	Arterial velocity
Family	3	2.95	4.79*	17.31*
Environment	1	4.20	0.28	0.02
Family × Environment	3	2.95	1.43	0.54
Error	28			

### Cardiac physiology

**Figure 3:**
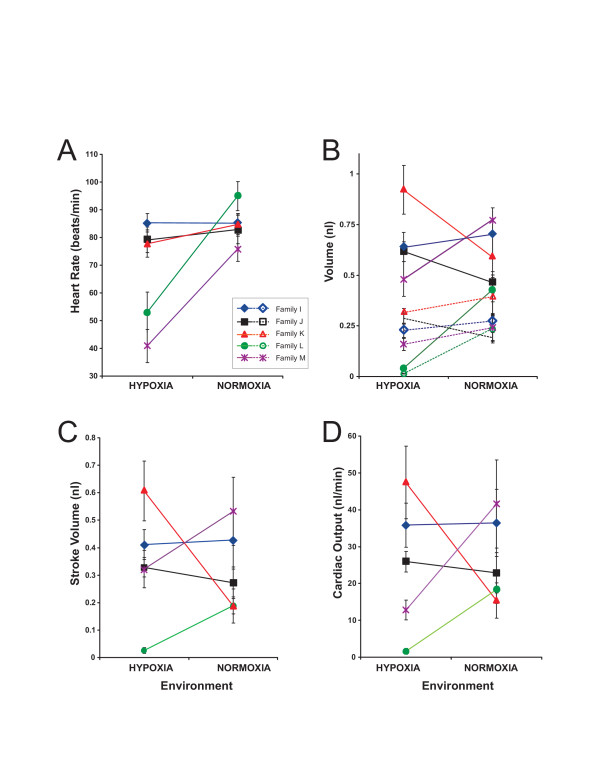
**Cardiac performance by family and environment**. Family means for; A) Heart Rate in beats/min, B) end systolic (open symbols) and diastolic (closed symbols) volume in nl, C) stroke volume in nl, and D) cardiac output in nl/min are presented for larvae reared in normoxic and hypoxic environments. Error bars represent one standard error. Family and Family × Environment interactions were significant sources of variation in all five traits, while a direct effect of Environment was only significant in heart rate and ESV.

Zebrafish family was also a highly significant contributor to variation in heart rate, end systolic volume (ESV), end diastolic volume (EDV), and stroke volume (SV) (Table 3). While developmental oxygen environment was a significant source of variation in heart rate and ESV, it did not significantly influence EDV, or SV (Table 3). Hypoxia-reared animals had lower heart rates and ESVs regardless of family (Figure 3A). Significant developmental environment by family interactions existed for heart rate, ESV, EDV, and SV (Table 3) indicating a strong family specific response to developmental oxygen level in these traits (Figure 3). Cardiac output (CO), which is an integrated measure of cardiac performance, varied highly significantly between families (Table 3). A significant Family × Treatment interaction term (Table 3) indicates that families differed in the degree and direction of response to developmental oxygen level (Figure 3). The cardiac response to oxygen environment was entirely family specific with no significant trend due to treatment (Table 3).

**Table 3:** Sources of variation in cardiac performance. ANOVA models were run using Family, Environment and Family by Environment interaction as the modelled sources of variation. Critical F values for the given degrees of freedom (DF) and the calculated F values are given for Heart Rate, End Systolic Volume (ESV), End Diastolic Volume (EDV), Stroke Volume (SV), and Cardiac Output (CO). Significant F values (p < 0.05) are in bold face and asterisks represent highly significant (p < 0.005) p values.

Source	DF	F value					
		
		Critical	Heart Rate	ESV	EDV	SV	CO
Family	4	2.63	8.93*	7.23*	10.27*	5.96*	4.37*
Environment	1	3.73	29.42*	**6.28**	0.88	0.12	0.26
Family × Environment	4	2.63	7.47*	**3.16**	5.54*	4.94*	5.42*
Error	38						

## Discussion

What processes constrain and redirect the outcome of the adult phenotype? We found that for cardiac output in larval zebrafish there is family specific developmental response to the environment (Table 3). While phenotypic development is clearly redirected by oxygen stress, this redirection is not consistent between families. These results indicate that genetic background is important in determining the cardiovascular system's developmental response to the environment. It is therefore important to investigate developmental response in multiple genetic backgrounds. From an evolutionary perspective, understanding the interplay between sources of variation in cardiac output response to the environment is critical to determining the degree to which that response is adaptive.

Plastic response to environmental variation is a major topic in evolutionary biology (for a discussion see [3,21,22]). In this study, plastic response of cardiac output was family specific, indicating that there is likely to be heritable variation for plastic response in cardiac performance. The significant family level variation in response to hypoxia in this study is the sum of parental environmental sources (e.g. "maternal effects") and genetic sources of variation. Variation in parental environment, however, was minimized due to the use of a common and tightly controlled environment (see methods). For this reason we believe that a large portion of the family level response that is observed in this study represents genetic variation.

Evolution of adaptive responses to variable environments requires heritable variation for plastic response. Given the proper selective environment, the variation in plastic response we find in cardiac output could be the raw material of future adaptation. To understand the dynamics of adaptation of a complex trait however, it is important to understand something about the way in which subsidiary traits contribute to that complex trait. If there are interacting traits that can combine in multiple ways to create optimal or near optimal response, then a rugged adaptive landscape will be produced [23,24] that will maintain variation by providing multiple genotypic targets for selection [25,26].

Cardiac output is the product of heart rate and stroke volume. Stroke volume is, in turn, the difference between end systolic and end diastolic volume. The family specific response to hypoxia for cardiac output could either be created by complex family specific interactions of these underlying traits, by family specific response in a single trait, or by a unified family specific response seen across all traits. Interestingly, all of the cardiac performance measures (Table 3) showed significant family specific responses to hypoxia. Furthermore, the family specific responses to hypoxia in heart rate (Figure 3A) and stroke volume (Figure 3C) do not correspond in all families (note opposing responses in family K but not other families). Likewise ESV and EDV show no consistent correspondence in their response to hypoxia. These families therefore clearly appear to be responding in a complex way creating a wide variation in integrated cardiac response. While one family responds to hypoxia with a 3 fold increase in cardiac output, another decreases cardiac output by an equivalent amount, and a third family remains constant across environments (Figure 3D). It is likely that there is a great deal of genetic variation in plasticity of cardiac output. In this case, however, selection on that plasticity should be constrained by the complex trait interaction leading to cardiac output. This is because selection will depend not on a single trait but on the variable interactions between traits [23,25].

While all cardiac performance traits show family specific responses to the environment, only heart rate and ESV showed a significant trend in the response to hypoxia (Table 3). When heart rate responded to hypoxia stress it was consistently in the direction of decreased contraction frequency (Figure 3A). Although tachycardia is the most commonly reported response to hypoxia[16], bradycardia has been documented in fish under chronic developmental hypoxia[20]. The trend was towards reduced ESV in hypoxia relative to normoxia (Figure 3). This weak trend is overshadowed by the clear lack of a consistent trend in stroke volume (Figure 3) to which it contributes.

Variation between families in cardiac performance in this study may be the product of both genetic and confounding maternal effects. While maternal effects on development can be important [4,27], there appear to be genetically predetermined physiological trajectories that embryos generally follow [6,9]. This study was not designed to distinguish between these two factors. Maternal effects themselves are the product of maternal genotype and environment. In this case, tight control of the parental environment makes it likely that maternal genetics account for a large proportion of any maternal effects that do occur in this study. Genetics, either directly or through maternal genetic influence, are therefore likely to play an important role in the family level variation documented in this study.

One curious finding is that, despite having the statistical power to detect small family differences in RBC velocity, oxygen environment did not influence RBC velocity either generally or in a family specific manner (Table 2). Given that considerable family specific environmental response in cardiac output occurs, it is at first surprising that corresponding changes are not found in RBC velocity. This may be due to an increase in arterial diameters (Table 1, Figure 1) that reduce the peripheral resistance to blood flow. In this case, this relatively small anatomical modification may ameliorate the effects of cardiac output changes on RBC flow rates. In addition, small increases in hematocrit, which was not measured, could increase blood viscosity also reducing red cell velocity. Increase in hematocrit in response to development in chronic hypoxia has been shown in zebrafish [28]. Finally, red blood cell velocity is also directly proportional to blood pressure. Without *in vivo *measurement of blood pressure, it remains unknown how pressure is altered by either environmental or family differences.

## Conclusion

Family-specific developmental responses to the oxygen environment play a major role in cardiac performance in zebrafish larvae. Genetic variation in plastic response to hypoxia may therefore provide the basic ingredient for adaptation to a variable oxygen environment via altered cardiac output. The complexity of physiological interactions that create that genetic variation may, however, slow the rate at which adaptation of plastic response to hypoxia should occur. While integrated cardiovascular response to hypoxia is family specific, red blood cell velocities were not altered by developmental environment at all, and arterial diameter changes in response to hypoxia were not family specific. It is therefore unlikely that these traits will evolve an adaptively plastic response to variable oxygen levels.

## Methods

### Animals

Zebrafish were chosen for this study because breeding pairs are easily manipulated, clutch sizes are large, fertilization and development is external allowing manipulation of the developmental environment and optical measurements through the transparent body wall can be made throughout early development [29]. Adult zebrafish (*Danio rerio*) were obtained from Scientific Hatcheries Inc. (Huntington Beach, CA) and populations of wild type and long fin gold strains were maintained as breeding stocks using standard husbandry procedures [30]. A 14L:10D light cycle and 26 ± 0.5°C water temperature was maintained throughout the study.

Males and female were paired randomly both within and between strains to produce larvae. The mating pairs were placed in 2 L containers supplied with a common water source (Z-Mod housing system, Marine Biotech, Beverly, MA) to minimize variation in the maternal environment. All larvae used were from the first clutch of eggs produced by a given female. By using only the first clutch for each female, we removed inter-clutch variation which could otherwise be a significant source of clutch level variation that is attributable to maternal, non-genetic environment [4]. We use the term family, or sibship to describe the relationship between the offspring for a single set of parents as is traditional in quantitative genetics. If the first clutch produced fewer than 60 eggs, the sibship was discarded and another parental pair was randomly matched. After a pair produced a clutch neither fish was used for further mating.

### Developmental treatment

Eggs from each clutch were removed before the eight-cell stage and randomly and equally divided into two groups. One group was placed into normoxic water (dissolved oxygen >6.0 mg O_2 _l^-1^) and the other group was placed into hypoxic water (dissolved oxygen 1.0 ± 0.2 mg O_2 _l^-1^). The system for housing the fish in both normoxic and hypoxic conditions is described in Marks et al. (2005). Briefly, the system is comprised of a partially split sump system (total volume of 1200 l) with O_2 _stripped from the hypoxic side of the sump by sparging with N_2 _in 2 m columns. Active transfer and diffusion allowed equilibration of water quality between treatments. The oxygen level and temperature were controlled by a YSI 5200 monitoring system.

### Measurements

#### Measurement techniques

At 96 hours post-fertilization, high-speed digital video recording was used for a series of morphological and physiological measurements. Larvae were placed in the treatment water in a temperature-controlled stage (Harvard Apparatus) under an inverted microscope (Leica DMIRB). Digital video was captured at 125 frames sec^-1^using a scope-mounted black and white high-speed digital video camera (Red Lake MASD, San Diego, CA). All measurements were made with the larvae in their own developmental oxygen and temperature environment.

#### Vessel diameter

Videos of trunk vessels were used to measure the diameter both of the dorsal and caudal vein and artery at the cross section of the anus (cloaca) as described in [29]. The actual diameter was found by drawing a straight line across the vessel using Image Pro^® ^software (Media Cybernetics, Silver Springs, MD). Each diameter was measured six times per larvae per location within one frame and the mean of these measures was used as the vessel diameter.

#### Red blood cell velocity

The velocity of red blood cells within the artery and vein was calculated from the high speed video files as described in [31]. Distance travelled in 0.008 seconds was calculated by tracing the distance one red blood cell moved during one frame of video recorded at 125 frames sec^-1^. The frame closest to the middle of a systolic cycle was used. Six distances were taken of six different red blood cells within one frame, and this procedure was repeated at six different frames in different beats to reduce sampling and measurement error.

#### Heart rate

Heart rate was measured by recording the time elapsed over 15 heart beats and extrapolating to beats per minute. This was measure was repeated six times per larvae and the mean heart rate was used as the individuals heart rate.

#### Stroke volume

To measure end systole volume (ESV) and end diastole volume (EDV), a single frame for each was chosen during the cardiac cycle, and the perimeter of the ventricle was outlined manually [9,31-33]. Six systoles and six diastoles were measured per larvae in order to reduce sampling error. The volumes were calculated by the equation:

(((8/3Π)*A^2^)/L) = Volume (nl)

with A representing area and L representing the length across the long axis. The calculation was performed on the mean values of each larva. To calculate stroke volume, ESV was subtracted from EDV.

#### Cardiac output

To obtain values for cardiac output, the values for mean stroke volume and mean heart rate for a given larvae were multiplied together. Thus,

Cardiac Output (nl/min) = Stroke Volume (nl) * Heart Rate (beats/min).

### Statistics

The purpose of this study was to determine if there is variation between families in the plastic response of the cardiovascular system to developmental oxygen levels. ANalysis Of VAriance (ANOVA) utilizing the general linear models procedure in the SAS software package (SAS Institute, Cary, NC) was used to test which, of several, sources of variation were significant contributors to variation in cardiovascular development. In these analyses, variance between family means would be indicated if Family was a significant source of variation. If developmental oxygen treatment (Environment) is a significant source of variation, it indicates that there is plasticity across environments. Testing for significant Family by Environment interaction (Family × Environment) allows detection of differences in the plastic response between families. In the absence of non-genetic maternal effects this term would comprise a portion of the Genotype × Environment term that represents the genetic variation in plastic response. Since examination of ANOVA residuals for violations of parametric assumptions did not indicate the necessity of any data transformations analysis was performed on untransformed data.

## Authors' contributions

MH participated in the design of the study, executed the experiments, collected and organized the data and produced a rough draft of the manuscript. FBGM participated in the design of the study, helped with its coordination, performed the statistical analyses and made a significant contribution to both the rough and the final draft of the manuscript. BB conceived of the study, participated in its design and coordination and helped in the drafting of both the rough and final copies of the manuscript. Manuscript revisions after reviewer comments were primarily completed by FBGM. All authors read and approved the final manuscript. FBGM and BB designed and constructed the environmental control systems.
